# Physicochemical properties and antioxidant activity of enzymatically grafted pullulan-ferulic acid conjugates

**DOI:** 10.1016/j.dib.2026.113046

**Published:** 2026-07-02

**Authors:** Koceila Boundaoui, Xavier Falourd, Corinne Loutelier-Bourhis, Tony Varacavoudin, Luc Picton, Didier Le Cerf, Virginie Dulong

**Affiliations:** a Univ Rouen Normandie, INSA Rouen Normandie, CNRS, PBS UMR 6270, F-76000 Rouen, France; b Univ Rouen Normandie, INSA Rouen Normandie, Univ Caen Normandie, ENSICAEN, CNRS, Institut CARMeN UMR 6064, F-76000 Rouen, France; c INRAE, UR1268 BIA, 44300, Nantes, France; d INRAE, CALIS/PROBE Research infrastructures, BIBS facility, 44300, Nantes, France

**Keywords:** Functional biomacromolecule, Polysaccharide, Green grafting, Self-association, Phenolic compounds

## Abstract

This article presents experimental data from the analysis of pullulan grafted with ferulic acid with low grafting rates via enzymatic catalysis. These data come from preliminary tests as described in the related research article “Antioxidant functionalization of pullulan with ferulic acid using enzymatic catalysis”. The Pull-FA derivatives’ grafting rates, measured by the Folin-Ciocalteu assay, are determined, as well as their UV signatures. These data supplement the main article and highlight the impact of the ferulic acid grafting on pullulan, even at low rates (between 3.1 and 9.7 mg.g^−1^). The various analyses carried out show the self-associative character of the derivatives even at low grafting rates. Finally, the products all exhibit antioxidant properties (analyzed using a DPPH• reagent). In summary, for Pull-FA-T40% having the highest FA content of 9.7 mg.g^−1^, the critical aggregation concentration was found at 0.13 g.L^−1^, and the radical scavenging reached 60%. The data may help for a better understanding of the behavior of naturally functionalized polysaccharides with phenolic compounds, as well as for those that are enzymatically or chemically modified.

Specifications Table

**Table:** 

Subject	Engineering & Materials science
Specific subject area	Enzymatic catalysis of polysaccharide functionalization with phenolic compounds
Type of data	Tables and Figures
Data collection	UV analyses, Folin-Ciocalteu assay, and antioxidant property (Cary 100 Bio spectrophotometer, Varian, USA). Size exclusion chromatography (two chromatography columns (OHPAK SB 804 and 806 HQ) preceded by a guard column (OHPAK SB-G) (Shodex Showa Denko K.K., Japan) equipped with online detectors: multi-angle light scattering (MALS) (DAWN Heleos-II, Wyatt Technology Inc., USA), differential refractive index (DRI) (RID 10A Shimadzu, Japan) and UV detector (SPD-M20A Shimadzu, Japan). Fluorescence measurements, Fluoromax-4 spectrophotometer equipped with a xenon lamp, Horiba Jobin Yvon, Japan. Viscometry with low-shear device, LS 400 Lamy Rheology, France.
Data source location	*Institution: University of Rouen Normandie, INSA Rouen Normandie, CNRS, PBS UMR 6270* *City: F-76000 Rouen* *Country: France*
Data accessibility	***Boundaoui, Koceila; Falourd, Xavier; Loutelier-Bourhis, Corinne; Varacavoudin, Tony; Picton, Luc; Le Cerf, Didier; Dulong, Virginie (2025), “Dataset for Data in Brief from Koceila et al (Physicochemical Properties and Antioxidant Activity of Enzymatically Grafted Pullulan-Ferulic Acid Conjugates)”, Mendeley Data, V1, doi:***10.17632/kvzk5zgnxf.1 Repository name: Dataset for Data in Brief from Koceila et al (Physicochem analysis of the enzymatically conjugated Pullulan with ferulic acid and the assessment of their antioxidant properties) Data identification number: ***doi:***10.17632/kvzk5zgnxf.1 Direct URL to data: https://data.mendeley.com/datasets/kvzk5zgnxf/1 Instructions for accessing these data: …
Related research article	Koceila BOUNDAOUI, Xavier FALOURD, Corinne LOUTELIER-BOURHIS, Tony VARACAVOUDIN, Luc PICTON, Didier LE CERF, Virginie DULONG, Antioxidant functionalization of pullulan with ferulic acid using enzymatic catalysis. BOUNDAOUI, K., FALOURD, X., LOUTELIER-BOURHIS, C., VARACAVOUDIN, T., PICTON, L., LE CERF, D., & DULONG, V. (2026). Antioxidant functionalization of pullulan with ferulic acid using enzymatic catalysis. Carbohydrate Polymers, 381, 125202. https://doi.org/10.1016/J.CARBPOL.2026.125202

## 1 Value of the Data

• These data are useful for understanding the physicochemical and functional behavior of neutral polysaccharides after enzymatic functionalization with low levels of phenolic compounds.
• These data provide an interesting basis for researchers for comparing enzymatically and chemically functionalized polysaccharides containing phenolic compounds at low grafting rates.
• The grafting rates reported in this study are similar to those naturally found in feruloylated arabinoxylans and pectins. Therefore, these data may help to better understand the natural behavior of these polysaccharides.
• The optimized reaction conditions caused the degradation of the macromolecular structure of pullulan. In contrast, the reaction conditions used to obtain the derivatives described in this article were non- or minimally degradative. Understanding the macromolecular properties reported here may help academic and industrial researchers select reaction conditions according to the desired final properties, including macromolecular structure, grafting rates, and antioxidant properties.

## 2 Background

We performed enzymatic grafting of ferulic acid onto pullulan to combine the macromolecular properties of polysaccharides with the functional properties of natural phenolic compounds. The aim was to produce covalently conjugated hybrid compounds with high added value for potential applications in the pharmaceutical, cosmetic, and agri-food industries. In addition, the use of a green enzymatic pathway avoids conventional chemical grafting methods and highlights the potential of enzymatic polymer catalysis. These data are related to the main article entitled ``Antioxidant functionalization of pullulan with ferulic acid using enzymatic catalysis'' [1]. They describe the chemical macromolecular, behavioral, and functional properties of low grafting rates derivatives that were not presented in the main article. The results demonstrate that ferulic acid grafting significantly affects pullulan properties, even at low grafting rates. Furthermore, the grafting rates of these derivatives are comparable to those reported for several enzymatic grafting systems in the literature [[2], [3], [4], [5]]. They are also similar to the natural grafting levels of phenolic compounds found in polysaccharides such as arabinoxylan [6] and pectin [7]. Therefore, these analyses provide a useful basis and contribute to a better understanding of the behavior of these functional polysaccharides (Table 1).

**Table 1:** List of the analyzed samples and names of the corresponding datasets.

Sample identification	Folin-Ciocalteu dataset	UV Analysis dataset	SEC/MALS/DRI/UV dataset	Fluorescence measurement dataset	Viscosimetry dataset	Antioxidant activity measurement dataset
Pull-FA-theoretical grafting rate (%)						
Pull	FC Pull	Pull	Pull	Pull X g L^−1^	Pull X g L^−1^	Pull + DPPH
FA	FA X g L^−1^	FA				
Pull-FA-T10%	Pull-FA-T10%	Pull-FA-T10%	Pull-FA-T10%			Pull-FA-T10% + DPPH; Pull-FA-T10% Blank
Pull-FA-T15%	Pull-FA-T15%	Pull-FA-T15%	Pull-FA-T15%			Pull-FA-T15% + DPPH; Pull-FA-T15% Blank
Pull-FA-T30%	Pull-FA-T30%	Pull-FA-T30%	Pull-FA-T30%			Pull-FA-T30% + DPPH; Pull-FA-T30% Blank
Pull-FA-T40%	Pull-FA-T40%	Pull-FA-T40%	Pull-FA-T40%	Pull-FA-T40% X g L^−1^	Pull-FA-T40% X g L^−1^	Pull-FA-T40% + DPPH; Pull-FA-T40% Blank
Pull-FA-T50%	Pull-FA-T50%	Pull-FA-T50%	Pull-FA-T50%			Pull-FA-T50% + DPPH; Pull-FA-T50% Blank
Pull-FA-T60%	Pull-FA-T60% Phase I and Phase II	Pull-FA-T60%	Pull-FA-T60%			Pull-FA-T60% Phase I + DPPH; Pull-FA-T60% Phase I Blank
Pull-FA-T100%	Pull-FA-T100% Phase I Phase II	Pull-FA-T100%	Pull-FA-T100%			Pull-FA-T100% Phase I + DPPH; Pull-FA-T100% Phase I Blank

## 3 Data Description

**Fig. 1:**
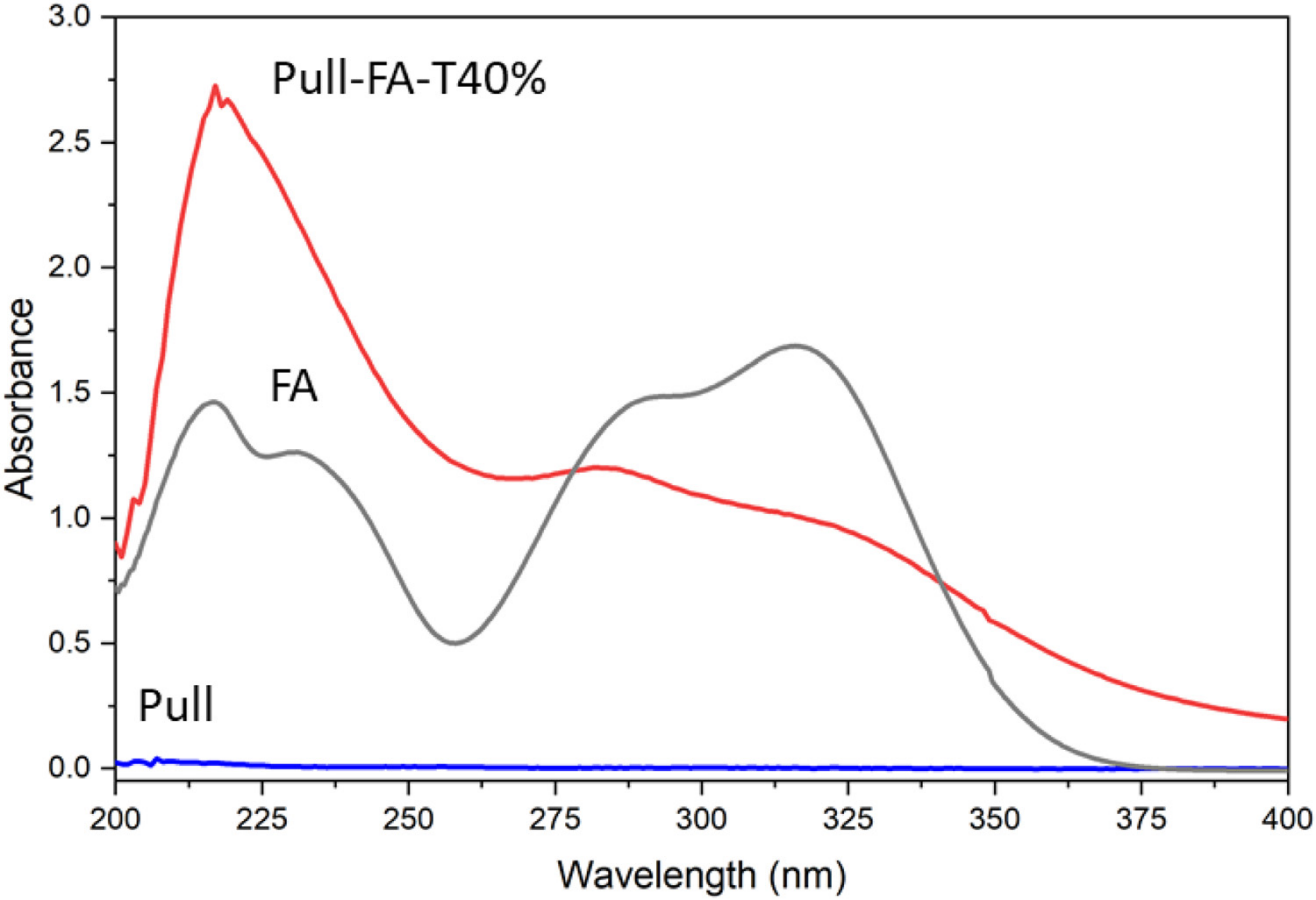
UV absorption spectra confirming successful FA grafting onto pullulan: UV absorption of Pull (blue), Pull-FA-T40% (red) at 1 g L^−1^ and free FA (grey) at 0.02 g L^−1^ in water.

**Fig. 2:**
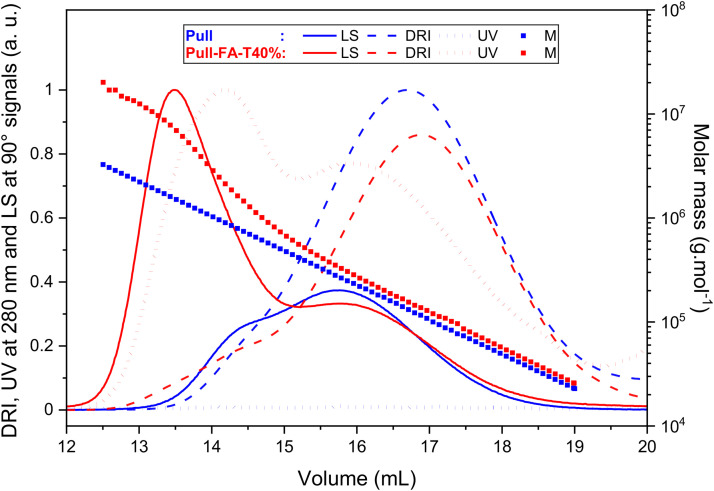
Determination of molar masses of Pull and Pull-FA-T40% by SEC/MALS/DRI/UV-280nm analysis and highlighting of the self-associative character Pull-FA-T40%. Pull (blue) and Pull-FA-T40% (red) at 2 g L^−1^ in filtered LiNO_3_ 0.1M at 25°C. Light scattering (LS) at 90°C (full line), refractive index (DRI) (dashed line), UV absorbance at 280 nm (dotted line), and calculated molar mass distribution (square).

**Fig. 3:**
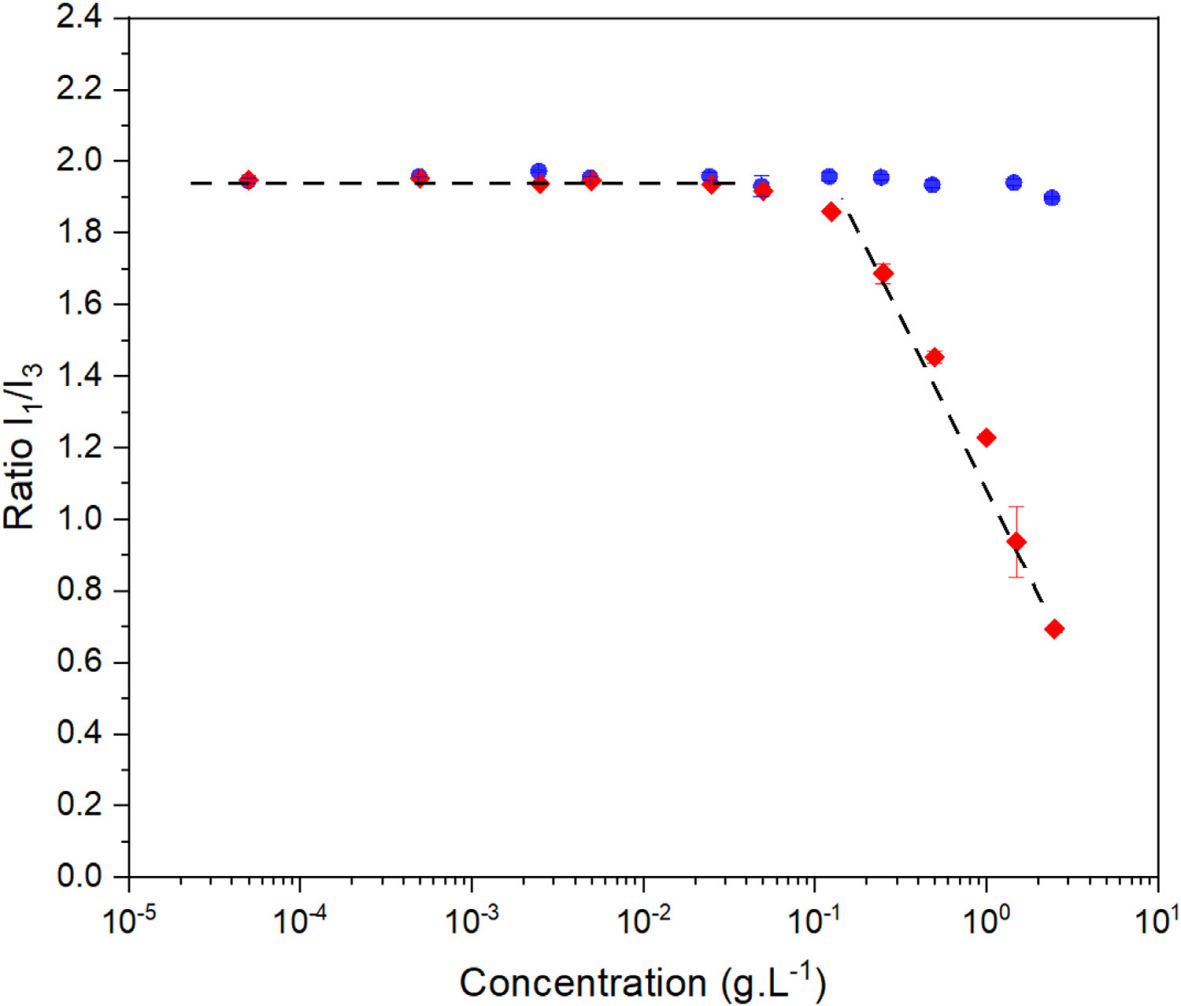
Fluorescence of pyrene showing the self-associative character of Pull-FA-T40%: critical aggregation concentration determination with I_1_/I_3_ ratio of pyrene fluorescence in water as a function of Pull (blue round) and Pull-FA-T40% (red diamond) concentration. (The critical aggregation concentration of Pull-FA-T40%, corresponding to the point where the lines crossed, was estimated at 0.13 g L^−1^).

**Fig. 4:**
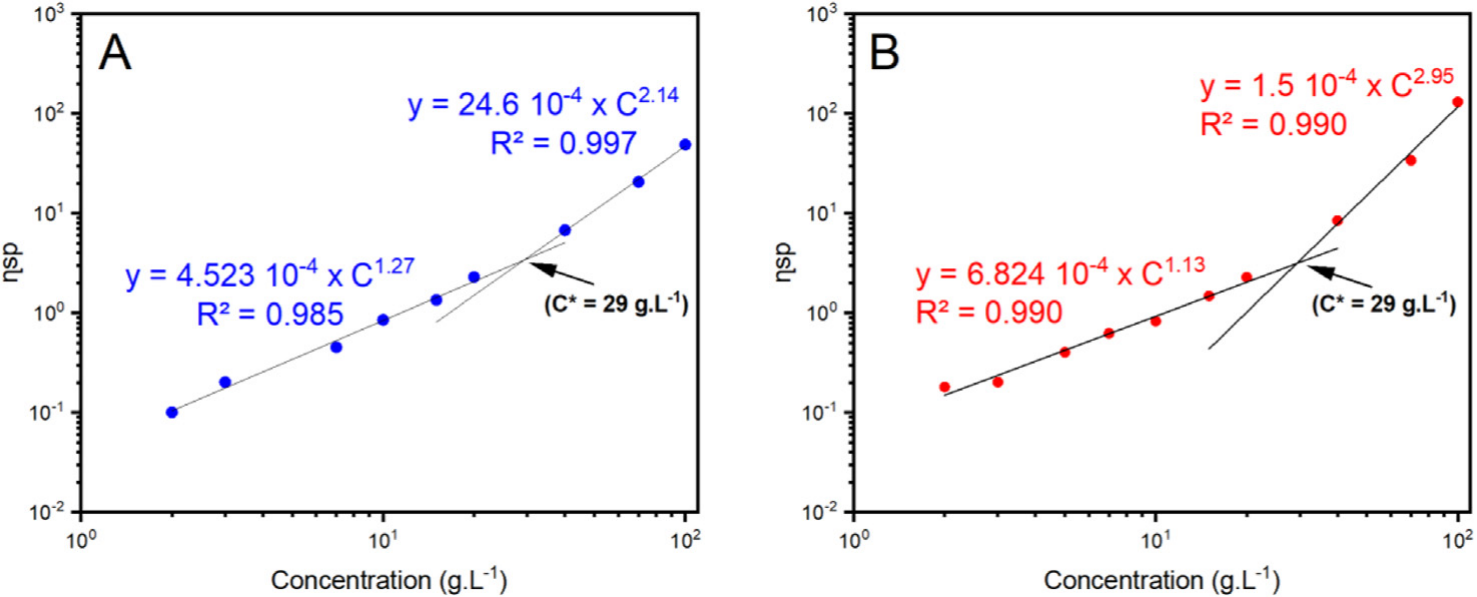
Determination of the critical concentrations of Pull and Pull-FA-T40%: specific viscosity evolution as a function of the concentration of Pull (blue) and Pull-FA-T40% (red) in water at 20°C.

**Fig. 5:**
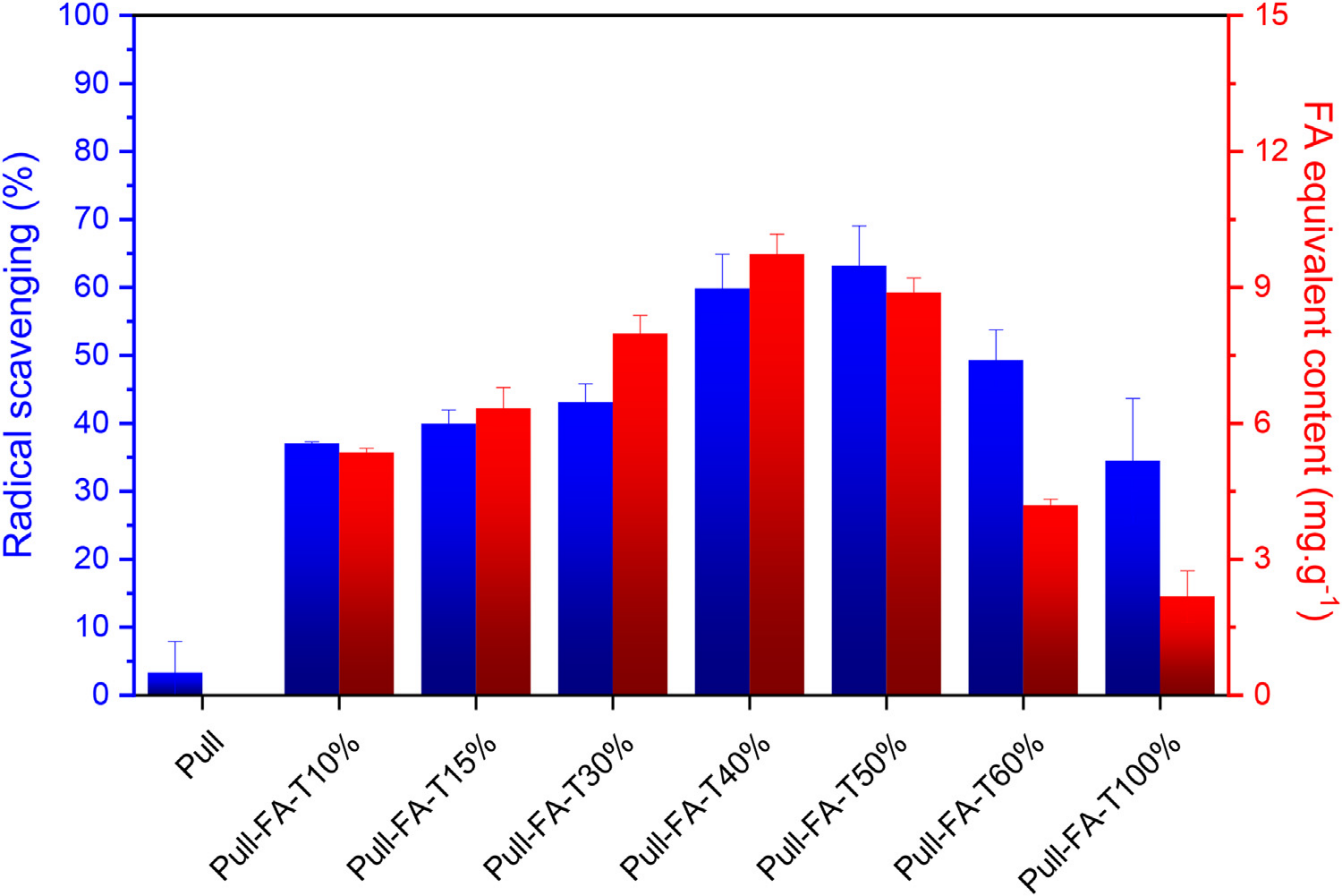
Radical scavenging activity in % of Pull and water-soluble Pull-FA showing the antioxidant character of derivatives at 7.5 g L^−1^ at 25°C estimated with DPPH^•^ (blue), compared to their estimated FA content in mg.g^−1^ with the Folin-Ciocalteu dosage (red).

These data were collected during a project aimed at increasing the added value of pullulan by introducing antioxidant phenolic compound, ferulic acid, in an eco-friendly manner. Several pullulan derivatives slightly grafted with ferulic acid were produced during the preliminary tests of the enzymatic reaction described in the main article. Herein, we present their grafting rates as determined by the Folin-Ciocalteu assay (Table 2). The phase I corresponds to the water-soluble product after purification and the phase II to the insoluble one. For theoretical grafting rates from 10 to 50% only phase I was observed (all the products were soluble in water). For theoretical grafting rates of 60% and 100% the two phases were present: a water-soluble one (phase I) and an insoluble one (phase II). The UV signature of Pull-FA-T-40% is shown in Fig. 1. Fig. 2 shows an example of a SEC/MALS/DRI/UV chromatogram, along with a summary table (Table 3) of the macromolecular parameters extracted from the different chromatograms (Mw, average molar mass in weight; Mn, average molar mass in number; Đ, dispersity; and Rg, radius of gyration). The self-associative effect of Pull-FAs was demonstrated through fluorescence measurements of the pyrene probe in dilute medium (Fig. 3) and viscosity measurements in semi-dilute medium (Fig. 4). Finally, antioxidant property measurements performed with the DPPH^•^ method are shown in Fig. 5.

**Table 2:** Experimental grafting ratio and efficiency of the different reaction products obtained by the Folin-Ciocalteu assay.

GR_T_ (%)	Phase I^b^			Phase II^c^		
	FA content (mg.g^-1^)	GR (%)	Efficiency (%)	FA content (mg.g^-1^)	GR (%)	Efficiency (%)
60^a^	≈ 0.0 ± 0.0	≈ 0.00 ± 0.00	-	-	-	-
10	5.4 ± 0.1	0.45 ± 0.01	4.5	-	-	-
15	6.3 ± 0.5	0.53 ± 0.04	3.5	-	-	-
30	8.0 ± 0.4	0.67 ± 0.03	2.2	-	-	-
40	9.7 ± 0.4	0.82 ± 0.04	2.1	-	-	-
50	8.9 ± 0.3	0.75 ± 0.03	1.5	-	-	-
60	6.0 ± 0.1	0.50 ± 0.01	0.8	15.3 ± 0.6	1.30 ± 0.05	2.2
100	3.1 ± 0.6	0.26 ± 0.05	0.3	16.7 ± 3.4	1.42 ± 0.29	1.4

GR_T_ theoretical molar grafting ratio of FA onto Pull

GR Experimental molar grafting ratio of FA onto Pull

FA content: X mg.g^-1^ X mg of the grafted FA per 1 g of the conjugated Pull-FA

a Blank reaction without laccase

b water-soluble phase

c insoluble phase

**Table 3:** Results obtained by SEC/MALS/DRI/UV for the Pull and the water-soluble Pull-FA.

Products	Mn (10^3^ g.mol^−1^)	Mw (10^3^ g.mol^−1^)	Đ	Rg (avg) (nm)	Mass recovery (%)
Pull	102 ± 2	209 ± 1	2.1 ± 0.0	25 ± 1	92
Pull-FA-T10%	112 ± 3	483 ± 2	4.3 ± 0.1	33 ± 1	90
Pull-FA-T15%	124 ± 3	655 ± 3	5.3 ± 0.1	35 ± 1	89
Pull-FA-T30%	103 ± 2	379 ± 2	3.7 ± 0.1	27 ± 1	83
Pull-FA-T40%	116 ± 2	511 ± 2	4.4 ± 0.1	31 ± 1	79
Pull-FA-T50%	107 ± 2	434 ± 2	4.1 ± 0.1	29 ± 1	63
Pull-FA-T60%	100 ± 2	364 ± 2	3.7 ± 0.1	30 ± 1	84
Pull-FA-T100%	108 ± 2	375 ± 2	3.5 ± 0.1	32 ± 1	81

## 4 Experimental Design, Materials and Methods

### 4.1 Materials

Pullulan (Pull) was provided by Hayashibara Biochemical Laboratory (Japan). Ferulic acid (FA), pyrene, and LiNO_3_ were purchased from Acros Organics (France). Laccase (EC 1.10.3.2) from *Myceliophthora thermophila* produced by *Aspergillus sp* genetically modified, Folin-Ciocalteu reagent, syringaldazine (SGZ), 2,2-Diphenyl-1-picrylhydrazyl (DPPH^•^) were purchased from Sigma Aldrich (France). Ethanol and anhydrous sodium carbonate were purchased from VWR. All compounds were used without further purification.

### 4.2 Synthesis protocol

The synthesis protocol was as follow: 0.5 g of Pull was solubilized in a double-necked flask overnight in 90 mL of phosphate buffer 0.1 mol.L^−1^, pH 7.5. Then, 10 mL of freshly solubilized FA in ethanol at equivalent concentrations to theoretical molar grafting ratios (GR_T_), which corresponds to the FA molar equivalent, expressed in percent, for one equivalent of anhydroglucose (Pull repeat sugar) ``10, 15, 30, 40, 50, 60, and 100%''. The mixture solution was stirred for 15 minutes before initiating the reaction with laccase addition (150 U, determined by the monitoring of the oxidation of syringaldazine by laccase at 25°C in a phosphate buffer 0.1 mol.L^−1^, pH 7.5.). Then, the mixture was stirred at 400 rpm for 24 hours without oxygen restriction at a thermostated temperature of 25°C. After that, the enzyme activity was stopped by thermal denaturation at 80°C for 35 min. The products were recovered as follow: acidification at pH 3.1 by HCl 1 mol.L^−1^; precipitation in acetone, then centrifugation at 4200 rpm (these two steps were repeated twice); solubilization of the products in Milli-Q water and dialysis against Milli-Q water using cut-off membranes of 12-14 kDa.

### 4.3 Folin-Ciocalteu assay protocol

The experimental protocol of Chan *et al.* was reproduced with slight modifications [8]. Pull and Pull-FA were solubilized at 10 g L^−1^ in Milli-Q water.

Briefly, 0.3 mL of the different solutions were placed in a test tube, followed by 1.5 mL of Folin-Ciocalteu reagent diluted 10 times in water. The mixture was then vortexed before adding 1.2 mL of carbonate solution at 75 g L^−1^. The solution was gently stirred for one hour using an orbital stirrer (Rotamax 120). Finally, the absorbance of the solution was measured at 750 nm using a Cary 100 Bio (Varian, USA) spectrophotometer. All measurements were performed in triplicate. The calibration curve was obtained with different concentrations of FA solutions ranging from 5.15×10^−6^ to 5.15×10^−4^ mol.L^−1^. The obtained slope of absorbance (Abs) as a function of FA concentration [FA] was expressed in equation (1). The FA equivalent content in the conjugate (mg.g^−1^ of Pull FA) was then calculated according to Eq. (2).

(1) Abs=1689.5×[FA]+0.0249withR²=0.999

(2) $$\text{FA}\;\text{content}=\frac{m_{\text{FA}}}{m_{\text{Pull}-\text{FA}}}\;\times \;1000=\frac{C_{\text{FA}}\;\times \;M_{\text{FA}\;}\times \;V}{m_{\text{Pull}-\text{FA}}}\;\times \;1000$$

C_FA_ and M_FA_ are the molar concentration of FA in the sample and its molar mass (194.18 g.mol^−1^), respectively. m_Pull-FA_ is the mass of the dry sample added and V is the volume of the sample solution added.

The expression of the experimental grafting ratio (GR) of FA onto Pull in equivalent mol of FA per 100 mol of anhydroglucose was obtained as follows:

(3) $$\text{GR}=\frac{n_{\text{FA}}}{n_{0\;\left(\text{Pull}-\text{FA}\right)}}\times 100=\frac{C_{\text{FA}}}{C_{0\;\left(\text{Pull}-\text{FA}\right)}}\times 100=C_{\text{FA}}\times \frac{M_{0\;\left(\text{Pull}-\text{FA}\right)}}{{C_{P}}_{\left(\text{Pull}-\text{FA}\right)}}\times 100$$

Where n_FA_ is the molar quantity of FA (in mole), C_FA_ the molar concentration of FA (in mol L^−1^). n_0 (Pull-FA)_ and C_0 (Pull-FA)_ are the average molar quantity (in mole) and molar concentration of the Pull-FA repeat unit (in mol L^−1^), whose molar mass is M_0 (Pull-FA)_ (in g mol^−1^) and C_p (Pull-FA)_ is the mass concentration of Pull-FA (in g L^−1^).

Knowing that the enzymatic grafting of phenolic compounds onto neutral polysaccharides according to this reaction was described as an etherification reaction, grafting induces a loss of 2 hydrogens. The expression of M_0 (Pull-FA)_ then becomes:

(4) $$M_{0\left(\text{Pull}-\text{FA}\right)}=M_{0\left(\text{Pull}\right)}+\frac{\text{GR}}{100}\times M_{\text{FA}}-2\times \frac{\text{GR}}{100}\;M_{H}=M_{0\left(\text{Pull}\right)}+\frac{\text{GR}}{100}\;\left(M_{\text{FA}}-2\;M_{H}\right)$$

Where M_o(Pull)_ is the molar mass of repeating unit of Pull (162 g mol^−1^), M_H_ the molar mass of hydrogen (1 g mol^−1^) and M_FA_ the molar mass of FA (194.18 g mol^−1^).

Replacing M_0(Pull-FA)_ with its expression in equation 3 gives the final equation for determining GR :

(5) $$\text{GR}=\frac{M_{0\left(\text{Pull}\right)}\times \frac{C_{\text{FA}}}{{C_{P}}_{\left(\text{Pull}-\text{FA}\right)}}}{1-\frac{C_{\text{FA}}}{{C_{P}}_{\left(\text{Pull}-\text{FA}\right)}}\times \left(M_{\text{FA}}-2\;M_{H}\right)}\times 100=\;\frac{162\times \frac{C_{\text{FA}}}{{C_{P}}_{\left(\text{Pull}-\text{FA}\right)}}}{1-\frac{C_{\text{FA}}}{{C_{P}}_{\left(\text{Pull}-\text{FA}\right)}}\times \left(194.18-2\right)}\times 100$$

### 4.4 UV-Vis analysis

A Cary 100 Bio (Varian, USA) spectrophotometer was used for UV-Vis analysis. Pull, FA and Pull-FA aqueous solutions were analyzed in the range of 200-400 nm to compare their UV spectra.

### 4.5 SEC/MALS/DRI/UV/Visco protocol

The macromolecular parameters of Pull and Pull-FA were analyzed at 25°C using size exclusion chromatography (SEC). The polymers were separated through a series of two chromatography columns (OHPAK SB 804 and 806 HQ) preceded by a guard column (OHPAK SB-G) (Shodex Showa Denko K.K., Japan). The stationary phase in the column was a controlled porous gel of poly(hydroxymethylmethacrylate). 0.1 mol.L^−1^ LiNO_3_ solution already filtered with a 0.1 µm filter unit (Millipore, USA) and degassed online (DGU-20A3 Shimadzu, Japan) was used as mobile phase (eluent) with a flow rate of 0.5 mL.min^−1^ (LC10Ai Shimadzu, Japan).

Solutions of freeze-dried samples (Pull and Pull-FA) were prepared at 2 g L^−1^ in eluent, then filtered at 0.45µm (Millipore, USA) before automatic injection of 100 µL in the device (SIL-20A, Shimadzu, Japan). The polymer was separated and then detected by different on line coupled detectors, which include a multi-angle light scattering (MALS) (DAWN Heleos-II, Wyatt Technology Inc., USA), a differential refractive index (DRI) (RID 10A Shimadzu, Japon), a UV detection at 280 nm (SPD-M20A Shimadzu, Japan) and a viscometer (Viscostar IV, Wyatt Technology, USA). The MALS detector was fitted with a K5 cell of 50 µL and 18 photodiodes (normalized relative to the 90° detector using bovine serum albumin). The concentration of each eluted fraction of the sample was estimated with the known value of dn/dC of 0.150 mL.g^−1^.

### 4.6 Critical aggregation concentration determination

Pyrene was used as a fluorescent probe with hydrophobic properties to assess the self-aggregation behavior of the Pull-FA. The experimental protocol was described in the literature [9]. In summary, 20 µL of a 2 10⁻³ mol.L^−1^ pyrene acetone solution was introduced into a flask. The acetone was then allowed to evaporate under ambient pressure and temperature conditions. Subsequently, 50 mL of water was added and the mixture was stirred overnight to obtain a pyrene water solution of 8 10^−7^ mol.L^−1^. Then, 1 mL of this pyrene solution was mixed with 1 mL of the sample water solution at concentrations ranging from 10^−4^ to 5 g L^−1^. After stirring overnight in the dark, measurements were performed using a Fluoromax-4 spectrophotometer (Horiba Jobin Yvon, Japan) equipped with a xenon lamp at an excitation wavelength of 332 nm and emission wavelength from 350 to 450 nm. All measurements were performed in triplicate.

### 4.7 Viscosity measurements

The apparent viscosity of Pull and Pull-FA-T40% solutions at 20°C and different concentrations ranging from 2 to 100 g L^−1^ was investigated using a Low Shear device (LS 400 Lamy Rheology, France) managed by Rheomatic software. Stainless steel mobile MS-LS 11 and measuring geometry MK-1 were used for the measurements. The flow of samples was determined by measuring the evolution of viscosity at the equilibrium according to the variation of shear rate from 1 to 10 s^−1^ to determine the Newtonian viscosity (ɳ_0_). The specific viscosity (${ɳ}_{\text{sp}}$) was also calculated according to the following Eq. (6).

(6) $$\eta_{sp}=\frac{\eta_{sample}-\eta_{solvent}}{\eta_{solvent}}$$

### 4.8 Antioxidant activity protocol

DPPH**^•^**, likely to capture hydrogen from antioxidant molecules, was used to quantify the antioxidant activity of the reagents and products. The protocol described in the literature was used with some adaptations [9]. Briefly, 0.5 mL of 400 ppm DPPH**^•^** solution in ethanol was added to 1.5 mL of a 10 g L^−1^ polymer water solution and left to stir in the dark for 40 min. The absorbance of the solutions was then recorded in triplicate from 400 to 800 nm. A DPPH^•^ control solution was also recorded under the same conditions without sample addition. The antioxidant power of the samples was then estimated directly from the decrease in DPPH^•^ maximum absorbance using Eq. (7).

(7) $$\text{Scavenging}\;\%=\frac{A_{\text{DPP}H^{•}}\;-A_{S}\;}{A_{\text{DPP}H^{•}}}\;\times \;100$$

$A_{DPPH^{•}}$ corresponds to the DPPH^•^ maximum absorbance and $A_{S}$ to the maximum absorbance of samples mixed with DPPH^•^ solution.

## Limitations

None.

## Ethics Statement

This research does not involve animal or human samples and therefore requires no ethical approval.

## CRedit Author Statement

**Koceila Boundaoui:** Writing - Original Draft, Conceptualization, Methodology, Validation, Investigation, Project administration; **Xavier Falourd:** Formal analysis; **Corinne Loutelier-Bourhis:** Formal Analysis; **Tony Varacavoudin**: Formal analysis; **Luc Picton**: Conceptualization, Methodology; **Didier Le Cerf:** Conceptualization, Methodology, Resources, Writing - Review & Editing, Supervision, Funding acquisition; **Virginie Dulong**: Conceptualization, Methodology, Resources, Writing - Review & Editing, Supervision, Funding acquisition

## Data Availability

Mendeley DataDataset for Data in Brief from Koceila et al (Physicochem analysis of the enzymatically conjugated Pullulan with ferulic acid and the assessment of their antioxidant properties) (Original data).
